# Comparison of remifentanil and esketamine in combination with propofol for patient sedation during fiberoptic bronchoscopy

**DOI:** 10.1186/s12890-023-02517-1

**Published:** 2023-07-11

**Authors:** Jia Nie, Wei Chen, Yu Jia, Yu Zhang, Haiying Wang

**Affiliations:** 1 Department of Anesthesiology, Affiliated Hospital of Zunyi Medical University, 149 Dalian Street, Zunyi, Guizhou 563000 P.R. China; 2 Guizhou Key Laboratory of Anesthesia and Organ Protection, Zunyi Medical University, Zunyi, Guizhou 563003 P.R. China

**Keywords:** Esketamine, Propofol, Remifentanil, Fiberoptic bronchoscopy, Sedation

## Abstract

**Background:**

Ideal sedation and analgesia strategies for fiberoptic bronchoscopy have not been found. At present, propofol based sedation strategy still has some defects, such as respiratory depression and blood pressure drop. It is difficult to meet the requirements of safety and effectiveness at the same time. The aim of this study was to compare the clinical efficacy of propofol/remifentanil with propofol/esketamine for patient sedation during fiberoptic bronchoscopy.

**Method:**

Patients undergoing fiberoptic bronchoscopy were randomly assigned to propofol/ remifentanil (PR group; *n* = 42) or propofol/esketamine (PK group; *n* = 42) for sedation and analgesia. The primary outcome was the rate of transient hypoxia (oxygen saturation (SpO_2_) < 95%). The secondary outcomes are the intraoperative hemodynamics, including the changes in blood pressure, heart rate, the incidence of adverse reactions, the total amount of propofol usage were recorded, and the satisfaction level of patients and bronchoscopists.

**Results:**

After sedation, the arterial pressure and heart rate of patients in the PK group were stable without significant decrease. Decreases in diastolic blood pressure, mean arterial pressure, and heart rate were observed in patients in the PR group (*P* < 0.05), although it was not of clinical relevance. The dosage of propofol in the PR group was significantly higher than that in the PK group (144 ± 38 mg vs. 125 ± 35 mg, *P* = 0.012). Patients in the PR group showed more transient hypoxia (SpO_2_ < 95%) during surgery (7 vs. 0, 0% versus 16.6%, *P* = 0.018), more intraoperative choking (28 vs. 7, *P* < 0.01), postoperative vomiting (22 vs. 13, *P* = 0.076) and vertigo (15 vs. 13, *P* = 0.003). Bronchoscopists in the PK group showed more satisfaction.

**Conclusion:**

Compared with remifentanil, the combination of esketamine with propofol in fiberoptic bronchoscopy leaded to more stable intraoperative hemodynamics, lower dosage of propofol, lower transient hypoxia rate, fewer incidence of adverse events, and greater bronchoscopists satisfaction.

## Introduction

In recent years, fiberoptic bronchoscopy has been widely used in the diagnosis and treatment of tracheal parenchymal diseases. The main indications of fiberoptic bronchoscopy are chronic cough of unknown cause, such as suspected bronchial tuberculosis and airway tumor [1]. In addition, atelectasis, pulmonary nodules and lumps, obstructive pneumonia, airway stenosis can be examined by bronchoscopy [2–4]. The etiological evidence for diagnosis can be obtained by bronchoscopic brushing and lavage. Tracheoscopy can be used in the treatment of bronchial foreign body removal, hemostasis of small bleeding site, and the treatment of intra-airway tumors, such as stenting, tumor resection, local radiotherapy and chemotherapy for lung cancer patients [5, 6].

Fiberoptic bronchoscopy need to be performed with medications that relieve spasm of the airway smooth muscle, dilate the airway, and reduce the secretion of airway glands. However, the most important drugs are sedatives and analgesics, which can eliminate airway reactions such as choking. Choking is caused by mechanical stimulation of the airway during surgery, such as fiber bronchoscopy contact of the glottis or airway induced choking. General anesthesia is very important in reducing the difficulty of examination operation and improving the safety, tolerance, and comfort of patients [7]. Therefore, it has become a focus of research in recent years to try different combinations of sedative and analgesic drugs to achieve safe and comfortable bronchoscopy [7–9]. Intravenous general anesthetic propofol, supplemented by midazolam (benzodiazepine sedative hypnotic) and fentanyl (opioid analgesic), is currently the most commonly used anesthesia combination for painless bronchoscopy [8]. The combination of remifentanil and propofol, which can provide both anesthesia and analgesia, is also one of the classic combinations. This combination has been widely used in anesthesia for short operations, such as gastroenteroscopy and fiber bronchoscopy [10, 11]. It has been reported that a remifentanil/propofol mixture provided effective sedation and rapid recovery in pediatric patients undergoing fiberoptic bronchoscopy [12]. In addition, remifentanil reduces the total duration of prolonged cough and was associated with better bronchoscopist satisfaction [13].

At the same time, because no tracheal intubation was performed during bronchoscopy examination, if the anesthesia was too deep, it will cause a passive situation for anesthetists and fiberoptic bronchoscope operators. Therefore, anesthesiologists have been exploring the optimal combination of anesthetic drugs to achieve appropriate deep sedation while providing analgesia. For example, Bermejo et al. found that moderate sedation with dexmedetomidine-remifentanil is safer than deep sedation with propofol-remifentanil for endobronchial ultrasound [14]. However, the inhibition of these drugs to respiratory and circulatory system functions is obvious, with certain risks of use.

Ketamine is a noncompetitive antagonist of the N-methyl-d-aspartic acid (NMDA) receptor and has sedative and analgesic properties when administered in subanesthetic doses [15]. Ketamine has been widely used in pediatric basic anesthesia because it has no significant inhibitory effect on respiration and has the advantage of mild hemodynamic stimulation effect [16, 17]. Esketamine is the dextral enantiomer of ketamine and is about twice as potent as ketamine [18]. Besides, compare to ketamine, esketamine has the characteristics of short recovery period, rapid cognitive recovery, and lower incidence of psychiatric side effects [17].

Clinical trials reported that esketamine can reduce bradycardia and hypotension caused by propofol while exerting analgesic effect [19]. Therefore, the combination of propofol and esketamine may improve the safety and comfort for patients undergoing fiberoptic bronchoscopy. The purpose of this randomized, double-blind study was to compare the anesthetic effects, hemodynamic variables, and satisfaction of bronchoscopists and patients of propofol + esketamine versus propofol + remifentanil during painless fiberoptic bronchoscopy.

## Methods

This is a prospective, randomized, double-blind trial comparing the safety and comfort in patients undergoing fiberoptic bronchoscopy with propofol/remifentanil versus propofol/ketamine. A total of 90 adult patients (aged 18–65 years; ASA grade I or II), who underwent selective fiberoptic bronchoscopy, were recruited in the study. Patients were randomly allocated into two groups with the random number table, with a 1:1 ratio and 42 cases in each group. Indications included diagnostic tests for tuberculosis (33%), tumor inspection (26%), pneumonia (25%), chronic cough (10%), and hemoptysis (6%). The exclusion criteria were dysfunction of liver, kidney and other organ, abnormal coagulation function, mental illness, severe respiratory insufficiency, and history of severe allergy to the test drugs. All included patients and the bronchoscopists who performs fiberoptic bronchoscopy were blinded to the test drugs. During fiberoptic bronchoscopy, the anesthesiologists are responsible for recording patient information, preparation of study drugs, and patient monitoring. Present study was approved by Ethics Committee of Affiliated Hospital of Zunyi Medical University (Reference number: KLL-2020–049), and registered in Chinese Clinical Trials Registry (registration number: ChiCTR2100048790). Informed consents of all included patients were obtained.

Patients received 4 ml of 2% aerosolized lidocaine before surgery. All patients received oxygen directly through a mask (2 L/min). All of the patients in the present study were ventilated with a nasopharyngeal ventilation tube. No neuromuscular blocker was used. Patients retained spontaneous breathing. All patients were monitored by electrocardiogram, SpO_2_, and noninvasive blood pressure measurements. Comparisons of changes in oxygen saturation during fiberoptic bronchoscopy between the remifentanil and esketamine groups before general anesthesia (Before), after giving of anesthetics and before nasopharyngeal tube insertion (Administration), after insertion of a nasopharyngeal ventilation tube (nasopharyngeal tube insertion), and at the end of examination (End of examination) have been made. Baseline readings were taken prior to sedation and then recorded until the end of surgery. Patients were randomly assigned to propofol/remifentanil group (PR group, *n* = 42) or propofol/remifentanil group (PK group, *n* = 42). Propofol 1.5 mg/kg and remifentanil 0.5 μg/kg for PR group or esketamine 0.2 mg /kg for PK group were injected intravenously. About 0.5–1 min after narcotics and analgesics administration, the patient lost their consciousness, and the bronchoscopy operation began after the patient's eyelash reflection disappeared.

During the operation, propofol can be given according to the reaction of patients in the two groups and the length of the examination to maintain the appropriate depth of anesthesia. The total dose of propofol administered during the operation was recorded. Adequate sedation was confirmed by bronchoscopists and anesthetist. The bronchoscopist assessed the satisfaction level to the general anesthesia immediately at the end of inspection. After surgery, when the patients were fully awake, they were asked to evaluate the satisfaction level to the anesthesia. Patients were also asked if they would request the same sedation method for future bronchoscopy (yes or no). The satisfaction of patients and bronchoscopists was assessed by an independent anesthesiologist without knowing the usage of drugs.

The primary outcome was the rate of transient hypoxia (SpO_2_ < 95%). The secondary outcomes were the intraoperative hemodynamics, including the changes in blood pressure, heart rate, the incidence of adverse reactions, the total amount of propofol usage were recorded, and the satisfaction level of patients and bronchoscopists. The sample size calculation was based according to SpO_2_. A pilot study of 20 patients from our hospital found that the rate of SpO_2_ < 95% in esketamine group was 0%, while the rate of SpO_2_ < 95% in remifentanil group was 19%, and this required a sample size of 39 per group to achieve a power of 90% and a type I error of 5%. To compensate for the possibility of dropout, we recruited 84 patients, 42 patients per group. Data were presented as mean ± SD. Student's t-test was used to analyze dose of propofol, and duration of sedation between the two groups. Chi-square test was used to analyze sex ratio of enrolled patients, the satisfaction level of patients and bronchoscopists, number of patients with adverse side-effects after anesthesia. Cardiopulmonary parameters were analyzed using an analysis of repeated measures of variance within groups and an inter-group student t-test. The results were considered statistically significant when *P* < 0.05.

## Results

A total of 90 patients were assessed for eligibility. Six patients, three in each group, were excluded from the trial due to high blood pressure, 84 patients were included in this study. The general information of the two groups, including age, sex, weight, height, and examination duration, showed no significant difference (Table 1).

**Table 1:** Basic characteristics of patients

**Characteristics**	**Remifentanil + propofol group (*n* = 42)**	**Esketamine + propofol group (*n* = 42)**	***P* value**
Sex (male/female)	21/21	21/21	1.000
Age (years, mean ± SD)	51.8 ± 9.6	49.8 ± 12.3	0.888
Height (cm, mean ± SD)	162.9 ± 4.6	162.6 ± 6.6	0.785
Weight (kg, mean ± SD)	54.8 ± 9.6	56.3 ± 11.4	0.749

**Fig. 1:**
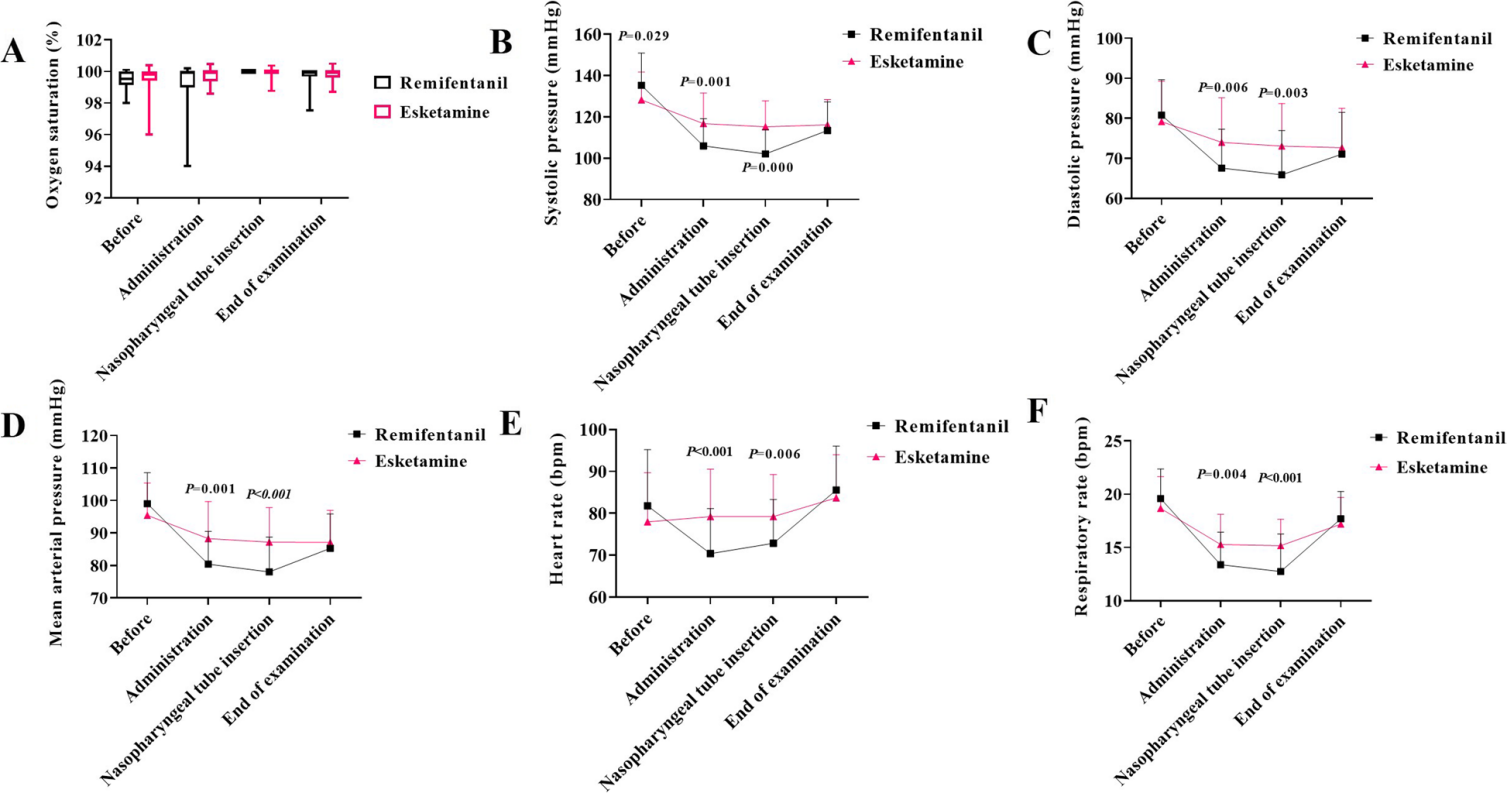
Changes in vital signs during fiberoptic bronchoscopy. The box-whisker plots show the comparison of changes between two groups before general anesthesia (Before), after giving of anesthetics and before nasopharyngeal tube insertion (Administration), after nasopharyngeal tube insertion (Nasopharyngeal tube insertion), and at the end of examination (End of examination). **A** Comparison of changes in oxygen saturation during fiberoptic bronchoscopy between the remifentanil and esketamine groups. **B** Comparison of changes in systolic pressure during fiberoptic bronchoscopy between the remifentanil and esketamine groups. **C** Comparison of changes in diastolic pressure during fiberoptic bronchoscopy between the remifentanil and esketamine groups. **D** Comparison of changes in mean arterial pressure during fiberoptic bronchoscopy between the remifentanil and esketamine groups. **E** Comparison of changes in heart rate during fiberoptic bronchoscopy between the remifentanil and esketamine groups. **F** Comparison of changes in respiratory rate during fiberoptic bronchoscopy between the remifentanil and esketamine groups. Data are mean ± SD. *n* = 42 in each group

The hemodynamic changes of the two groups during the operation were shown in Table 2 and Fig. 1. After sedation, the arterial pressure and heart rate of patients in the PK group were stable without significant decrease. Decreases in diastolic blood pressure, mean arterial pressure, and heart rate were observed in patients in the PR group. No intraoperative intervention for hypotension was given in the two groups.

**Table 2:** Changes in vital signs of patients during fiberoptic bronchoscopy (mean ± SD)

**Characteristics**	**Remifentanil + propofol group (*n* = 42)**	**Esketamine + propofol group (*n* = 42)**	***P* value**
SBP, mmHg			
Pre-administration	135.3 ± 15.6	129.2 ± 13.5	0.029
After administration	106.0 ± 13.2	116.8 ± 14.8	0.001
After asopharyngeal tube insertion	102.1 ± 11.7	115.3 ± 12.5	0.000
At end of examination	113.4 ± 13.9	116.2 ± 12.3	0.337
DBP, mmHg			
Pre-administration	80.8 ± 8.80	79.2 ± 10.09	0.405
After administration	67.6 ± 9.7	74.0 ± 11.1	0.006
After asopharyngeal tube insertion	65.9 ± 11.0	73.1 ± 10.6	0.003
At end of examination	71.1 ± 10.4	72.6 ± 9.8	0.467
MAP, mmHg			
Pre-administration	99.0 ± 9.6	95.4 ± 10.0	0.056
After administration	80.4 ± 10.1	88.3 ± 11.4	0.001
After asopharyngeal tube insertion	78.0 ± 10.7	87.2 ± 10.6	0.000
At end of examination	85.3 ± 10.6	87.1 ± 9.9	0.415
HR, bpm			
Pre-administration	81.8 ± 13.4	78.0 ± 11.8	0.289
After administration	70.4 ± 10.8	79.2 ± 11.4	0.000
After asopharyngeal tube insertion	72.9 ± 10.4	79.2 ± 10.0	0.006
At end of examination	85.6 ± 10.5	83.7 ± 10.3	0.415
RR, bpm			
Pre-administration	19.6 ± 2.8	18.7 ± 3.0	0.145
After administration	13.4 ± 3.07	15.3 ± 2.8	0.004
After asopharyngeal tube insertion	12.7 ± 3.5	15.2 ± 2.5	0
At end of examination	17.7 ± 2.6	17.2 ± 2.5	0.411

*SBP* systolic pressure, *DBP* diastolic pressure, *MAP* mean arterial pressure, *HR* heart rate, *RR* respiratory rate

**Fig. 2:**
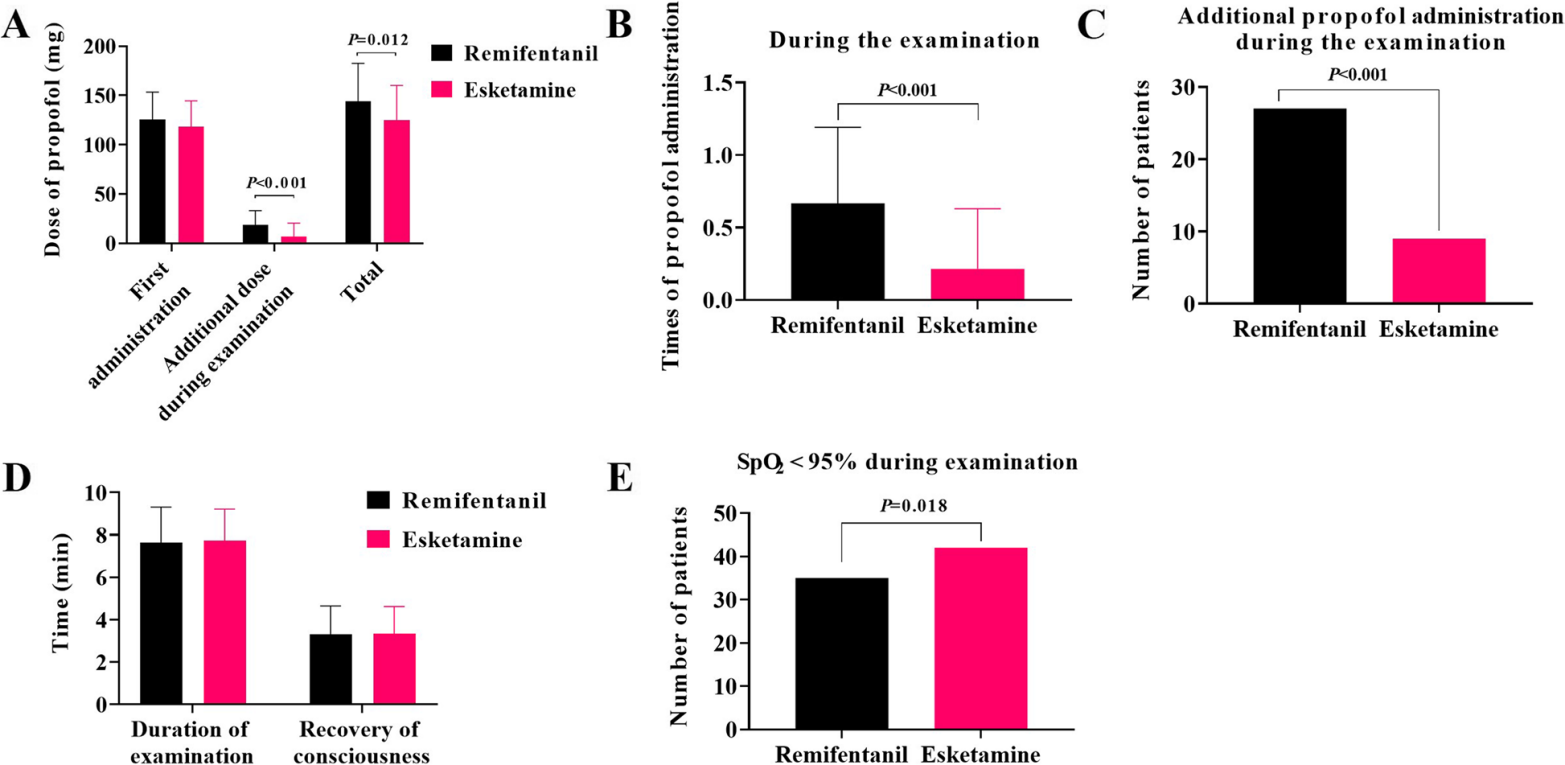
Comparison of propofol use and Ramsay scores during fiberoptic bronchoscopy.** A** Comparison of the dose of propofol used at first administration, additional dose of propofol, and total dose of propofol between the remifentanil and esketamine groups. **B** Comparison of the times of additional propofol administration during fiberoptic bronchoscopy between the remifentanil and esketamine groups. **C** The number of patients used additional propofol during fiberoptic bronchoscopy in each group. **D** Comparison of duration of fiberoptic bronchoscopy and time of recovery of consciousness after anesthesia between the groups. **E** Comparison of the number of patients with oxygen saturation below 95% during fiberoptic bronchoscopy

SpO_2_ did not decrease significantly in both groups before and after administration and during the fiberoptic bronchoscopy operation, and there was no significant difference of SpO_2_ between the two groups (Table 2). The total amount of propofol used during surgery was 144 ± 38 mg in the PR group and 125 ± 35 mg in the PK group. The consumption of propofol in the PR group was significantly higher than that in the PK group (*P* = 0.012, Table 3, Fig. 2). The number of patients in the PR group requiring additional propofol was significantly higher than that in the PK group (27 vs. 9, *P* < 0.001, Table 3, Fig. 2). In the PK group, all patients had no transient hypoxia during surgery (SpO_2_ < 95%), which was less than that of the PR group (0 versus 7, *P* = 0.018, Table 3, Fig. 2), although no patient showed SpO_2_ value < 90%. There was no significant difference in fiberoptic bronchoscopy operation time and consciousness recovery time between the two groups.

**Table 3:** Comparison of the use of propofol during fiberoptic bronchoscopy (mean ± SD)

**Characteristics**	**Remifentanil + propofol group (*n* = 42)**	**Esketamine + propofol group (*n* = 42)**	***P* value**
Dose of propofol used at first administration, mg	125.6 ± 27.8	118.0 ± 26.5	0.201
Number of patients used additional propofol, yes/no	27/15	9/33	0.000
Additional dose of propofol, mg	18.8 ± 14.5	6.9 ± 13.7	0.000
Times of giving additional propofol	0.7 ± 0.5	0.2 ± 0.4	0.000
Total dose of propofol, mg	144.4 ± 38.2	125.0 ± 35.3	0.012
Number of patients with SpO_2_ < 95%	35/7	42/0	0.018
Duration of examination, min	7.6 ± 1.7	7.7 ± 1.5	0.614
Recovery of consciousness, min	3.1 ± 1.3	3.4 ± 1.3	0.857

There were no major complications. The number of patients with intraoperative choking (28 vs. 7, *P* < 0.01, Table 4), postoperative vomiting (22 vs. 13, *P* = 0.076, Table 4), and vertigo (15 vs. 13, *P* = 0.003, Table 4) in PR group was significantly higher than that of PK group.

**Table 4:** Comparison of adverse reactions and satisfaction of patients and doctors during fiberoptic bronchoscopy

**Characteristics**	**Remifentanil + propofol group (*n* = 42)**	**Esketamine + propofol group (*n* = 42)**	***P* value**
Number of patients chocked after nasopharyngeal tube insertion	14	35	< 0.001
Number of patients with vomiting after examination	20	29	0.076
Number of patients with vertigo after examination	27	29	0.003
Number of patients with nausea after examination	38	41	0.356
Satisfaction level of patients to the operation			/
Excellent	42	42	
Good	0	0	
Poor	0	0	
Satisfaction level of bronchoscopists to the anesthesia			< 0.001
Excellent	15	32	
Good	14	10	
Poor	13	0	

Most patients in both groups were assessed as moderately sedated, and three patients in the PK group were over-sedated, however, no case was terminated prematurely due to excessive sedation. Thirty minutes after completion of bronchoscopy, all patients were fully recovered compared to their condition prior to sedation. The satisfaction level feedback attributed by patients was not significantly different in the two groups (Table 4). The bronchoscopists were more satisfied with the anesthesia outcomes in PK group than those in the PR group (Table 4). In PR group, the sedation level of 13 patients were considered poor by bronchoscopist (Table 4).

## Discussion

In the present study, esketamine combined with propofol provided less transient hypoxia, better hemodynamic stability and improved bronchoscopists satisfaction for fiberoptic bronchoscopy, compared with remifentanil group.

Propofol is the basic anesthetic for sedative and painless bronchoscopy, but when used alone, relatively large doses of propofol are required to achieve adequate sedation. High dose of propofol may cause hypotension or respiratory depression [20]. Remifentanil or ketamine combined with propofol can reduce the dose and side effects of propofol [21–23]. Ketamine can activate the sympathetic nerve and has the function of keeping the airway open and stimulating breathing [24]. Esketamine, the newly marketed S-enantiomer of ketamine, has enhanced anesthetic effect [25] and reduced incidences of psychiatric side effects of ketamine [26].

Therefore, we would expect esketamine to reduce oxygen desaturation and choking more than remifentanil during painless bronchoscopy. However, there was no significant difference between the two groups in terms of mean minimum oxygen saturation, and only esketamine was found to reduce the incidence of intraoperative choking. We believe that this is because esketamine has sedative and general anesthesia effects, therefore, patients have a deeper depth of general anesthesia after using esketamine, which can inhibit their cough reaction. While remifentanil has no sedative or general anesthetic effect. Remifentanil is an opioid that has a theoretical choking suppressant effect [27]. The addition of intravenous opioids to sedation regimens reduces choking during bronchoscopy [28]. In the current study, neither the combination of esketamine nor remifentanil eliminated intraoperative choking, but patients in both groups were well able to tolerate nasopharyngeal tube insertion. Because the patients in this study received ventilation through nasopharynx ventilation catheter, transient SpO_2_ decrease occurred in both groups during examination, may be due to reflex bronchoconstriction response, persistent choking, or endobronchial aspiration.

The primary outcome was the rate of transient hypoxia (SpO_2_ < 95%). The secondary outcomes were the intraoperative hemodynamics, including the changes in blood pressure, heart rate, the incidence of adverse reactions, the total amount of propofol usage were recorded, and the satisfaction level of patients and bronchoscopists. Our data showed that MAP and HR at nasopharyngeal tube insertion time point in the esketamine group were higher than those in the remifentanil group. However, MAP and HR at nasopharyngeal tube insertion time point decreased significantly in remifentanil group compared with administration time point, while MAP and HR in esketamine group were stable without significant fluctuation, which may be related to the cardiovascular excitation effect of esketamine [29, 30]. At the insertion time point of bronchoscopy, the elevated level of MAP and HR in remifentanil group was significantly higher than that in esketamine group, indicating that esketamine provided better circulative stability. Similar to this study, Hegazy et al. reported that the combination use of fentanyl and propofol induced hemodynamics change [31], while the combination use of ketamine and propofol led to stable hemodynamics [32]. However, neither remifentanil combination nor esketamine combination completely prevented the increase in blood pressure and heart rate caused by nasopharyngeal tube insertion, but such increase in blood pressure and heart rate was well tolerated and did not require any intervention. In addition, lower arterial pressure in the remifentanil group is statistically significant compared with esketamine group, however, it is not necessarily of clinical relevance.

The total dosage of propofol in the esketamine group was 125 ± 35 mg, which was lower than the 144 ± 38 mg in the remifentanil group. More patients needed additional propofol in remifentanil group, however, there was no significant difference in the recovery time between the two groups after examination. These results indicated that the combination of propofol and esketamine could reduce the dosage of propofol use without affecting the depth of general anesthesia. A clinical study on cholangiopancreatography also found that the combination use of propofol and esketamine could reduce the total amount of propofol required without affecting the time to recovery [33].

In the present study, the incidence of adverse events in 2 groups was also observed. Among which 7 cases of choking (16.67%) and 13 cases of postoperative vomiting (30.95%) in esketamine group were significantly less than 28 cases of choking (66.67%) and 22 cases of postoperative vomiting (52.38%) in remifentanil group. In addition, in esketamine group, patients had no transient hypoxia during surgery (SpO_2_ < 95%), compared with that of the PR group (0 versus 7, 0% versus 16.6%, *P* = 0.018)”. This is statistically significant and clinically relevant. It indicates that the respiratory inhibitory effect of esketamine is lighter than remifentanil at the same anesthesia depth. None of the patients in the esketamine group reported hallucination. The most mentioned ketamine-related side effect is delirium or hallucinations. This is more common if ketamine is used as the only sedative. In the present study, no patients reported delirium or hallucinations, which may be because the dose (0.2 mg /kg) of esketamine is low, or propofol eliminates the delirium or hallucinogenic side effects of esketamine [34].

The limitations of this study include small sample size, and only evaluated the effects of 0.2 mg /kg esketamine in fiberoptic bronchoscopy, further study need to be done to explore the best dose of esketamine for combination with propofol in fiberoptic bronchoscopy.

In conclusion, compared with remifentanil, esketamine combined with propofol provided more stable hemodynamics, improved the quality of early postoperative recovery, and reduced the incidence of adverse events, such as transient hypoxia, during fiberoptic bronchoscopy.

## Data Availability

The datasets used and analyzed during the current study are available from the corresponding author on reasonable request.
